# Rational Design, Synthesis, and Biological Assessment of Potential Indole‐Capped HDAC6 Inhibitors for Gastric Cancer Suppression

**DOI:** 10.1002/mco2.70158

**Published:** 2025-03-20

**Authors:** Ya Gao, Hai‐Qian Nie, Hong‐Min Liu, Xin‐Hui Zhang, Li‐Ying Ma

**Affiliations:** ^1^ State Key Laboratory of Esophageal Cancer Prevention & Treatment; Key Laboratory of Advanced Drug Preparation Technologies, Ministry of Education of China; Key Laboratory of Henan Province for Drug Quality and Evaluation, Henan Province; Institute of Drug Discovery and Development; School of Pharmaceutical Sciences Zhengzhou University Zhengzhou Henan China; ^2^ School of Biological Engineering Henan University of Technology Zhengzhou Henan China

1

Dear Editor,

Gastric cancer and gastroesophageal junction cancer have long been among the top killers in the cancer realm [1]. Moreover, East Asian countries such as China, Japan, and South Korea are known as high‐incidence regions for gastric cancer on a global scale. As the limited availability of precision medicine drugs for gastric cancer at present, there is still a need for the development of small molecule compounds targeting novel targets. Accumulating evidence suggests a link between dysregulated histone deacetylase 6 (HDAC6) activity and numerous oncological disorders [2, 3]. HDAC6 is frequently overexpressed in various malignancies, including primary acute myeloid leukemia, gastric cancer, breast cancer, ovarian cancer, and colorectal cancer, and is strongly associated with unfavorable prognosis [4]. These findings underscore the pivotal role of HDAC6 as a prominent therapeutic target in the pathogenesis and progression of diverse tumor types.

To develop small molecule inhibitors targeting the HDAC6 enzyme, we relied on the in‐house library compounds screening strategy and obtained a lead compound. We then optimized the structure of the lead compound **L9**. Among them, enzyme activity assays revealed compound **10n** exhibited excellent inhibitory effects on HDAC6 with an IC_50_ value of 3.11 nM. Simultaneously, **10n** showed notable selectivity in inhibiting HDAC1, HDAC2, HDAC3, and HDAC8 compared to HDAC6, with selectivity ratios of 133.7‐fold, 27.8‐fold, 82.8‐fold, and 622.22‐fold, respectively. Ricolinostat (ACY‐1215), one selective HDAC6 inhibitor in the clinical stage, exhibited IC_50_ values of 50, 42, 60, 120, and 5.3 nM for HDAC1, 2, 3, 6, and 8, respectively. The selectivity ratios of HDAC1/6, 2/6, 3/6, and 8/6 are 9.4‐fold, 7.9‐fold, 11.3‐fold, and 22.6‐fold, respectively. Compound **10n** exhibits greater selectivity against HDAC1, HDAC2, HDAC3, and HDAC8 compared to HDAC6. This indicates that **10n** is a significantly selective HDAC6 inhibitor.

*S*ilico docking study revealed that **10n** was capable of fitting into the substrate‐binding pocket of HDAC6. Additionally, the C═O group was seen to form a hydrogen bond with a Zn^2+^‐bound water molecule, with as O**^…^** H separation of 2.05 Å (Figure 1A). Furthermore, the N‐benzylformamide‐based linker portion nested in the hydrophobic tunnel and was sandwiched between P583 and P643, forming a clear π–π stacking interaction with P643 (Figure 1A). Additionally, BLI verified that **10n** exhibits a high affinity for HDAC6, with a Kd value of 330 ± 180 nM (Figure 1B).

**FIGURE 1:**
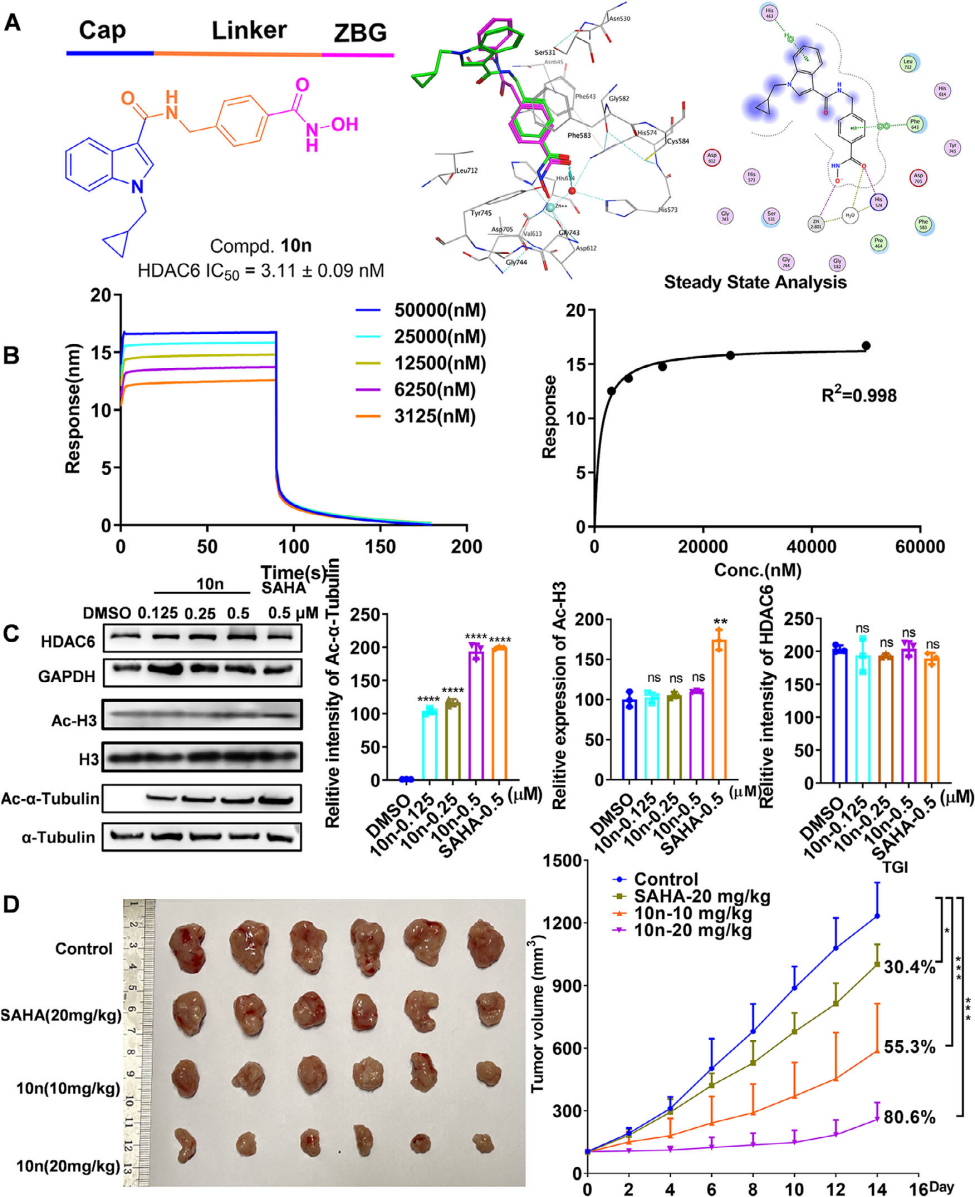
Compound **10n** exhibits antigastric cancer activity both in vivo and in vitro by inhibiting HDAC6 activity. (A) Docking pose of **10n** bound into the active pocket of zebrafish HDAC6 (PDB code 5EF7, 1.9 Å). (B) BLI binding of **10n** to HDAC6. (C) Analysis of the levels of HDAC6, Ac‐H3, and Ac‐α‐tubulin by western blot. Data are representative of three independent experiments. (D) Tumor image (*n* = 6) and changes in tumor volume over the course of treatment with vehicle control, **10n** (10 mg/kg or 20 mg/kg) or SAHA (20 mg/kg) in vivo. ****p < 0.0001, ***p < 0.001, **p < 0.01, *p < 0.05 compared with the control group.

Furthermore, **10n** exhibited enhanced cytotoxicity against MGC‐803 and MKN‐45 cells, with IC_50_ values of 1.067 ± 0.312 and 2.704 ± 0.935 µM at 72 h. EdU incorporation assays and plate clone formation assays were conducted to evaluate the antiproliferative effects of **10n**. Upon treatment with **10n**, there was a dose‐dependent increase in the proportion of apoptotic cells and an increase in mitochondrial membrane potential ratios. Consistent with expectations, **10n** could dose‐dependently induce the expressions of the PARP, Caspase 3, and Caspase 9, while the expressions of active form cleaved‐PARP, cleaved‐Caspase 3, and cleaved‐Caspase 9 were significantly decreased when cells were exposed to **10n**. These findings indicated that **10n** induced pro‐apoptotic effects through mitochondrial‐dependent apoptosis in gastric cancer cells.

Moreover, the interaction between **10n** and HDAC6 within cellular environments was conducted. **10n** notably stabilized HDAC6, suggesting the binding capability of **10n** to HDAC6 at the cellular level by CETSA. Additionally, **10n** induced a dose‐dependent increase in acetylated α‐tubulin levels while having no impact on acetylated H3 levels and HDAC6 (Figure 1C). The expression of PD‐L1 acts as a pivotal prognostic marker for the responsiveness of antitumor T cell immunity [5]. It is noteworthy that the PD‐L1 expression in MGC‐803 cells exhibited a dose‐dependent reduction in the **10n** treatment cohort compared to the vehicle control group, potentially enhancing the antitumor immune response in gastric cancer cells.

After characterizing the antigastric cancer cell activity of **10n** in vitro, a pharmacokinetic study was performed in mice. **10n** exhibited a good half‐life of 1.61 ± 0.34 h (i.v.) and 2.00 ± 0.44 h (p.o.), demonstrating acceptable systemic exposure with an area under the curve (AUC) value of 349.61 ± 51.93 h ng/mL (i.v.) and 729.98 ± 139.74 h ng/mL (p.o.). The oral bioavailability was calculated to be 41.8 ± 0.08%. Given these favorable pharmacokinetic parameters, **10n** was selected for subsequent in vivo antitumor experiments. As indicated in Figure 1D, **10n** significantly inhibited the growth of MFC cells at oral doses of 10 mg/kg and 20 mg/kg, achieving tumor growth inhibition rates (TGI) of 55.3% and 80.6%, respectively, surpassing that of pan‐HDAC inhibitor SAHA. Notably, the body weight of all mice steadily increased, suggesting its minimal toxicity. Consistent with the in vitro results, treatment with **10n** markedly elevated the levels of Ac‐α‐tubulin with no changes in HDAC6 protein expression were observed in animals treated with **10n**. Furthermore, Ki‐67 expression decreased upon **10n** administration in mice. Histopathological examination using H&E staining demonstrated that **10n** exerts a pro‐apoptotic function, thereby inhibiting the progression of gastric cancer. The percentages of CD3^+^CD8^+^ cells in gastric cancer tumors from two treatment groups were higher than the control. Furthermore, the **10n**‐treated group exhibited no apparent toxicity, as evidenced by stable weight and absence of organic alterations.

In this study, we identified a novel HDAC6 inhibitor, **10n,** which shows the strongest inhibitory effect on HDAC6 with an IC_50_ value of 3.11 ± 0.09 nM and a high binding affinity for HDAC6 (Kd = 330 ± 180 nM). Subsequent studies have substantiated the significant impact of **10n** in inducing apoptosis and alleviating immunosuppression, leading to the inhibition of gastric cancer cells both in vitro and in vivo. Additionally, **10n** exhibited promising pharmacokinetic characteristics and showed no significant side effects in vivo. These findings underscore the potential of compound **10n** as a promising therapeutic intervention for impeding gastric cancer advancement (Supporting Information).

## Author Contributions

**Ya Gao**: conceptualization, funding acquisition, investigation, methodology, supervision, writing – original draft. **Hai‐Qian Nie**: investigation. **Hong‐Min Liu**: conceptualization, funding acquisition. **Xin‐Hui Zhang**: conceptualization, writing – review & editing, funding acquisition. **Li‐Ying Ma**: conceptualization, writing – review & editing. All authors have read and approved the final manuscript.

## Ethic Statement

The animal experimentation ethics number is ZZU‐URIB‐2024‐O‐109. All experimental animals adhered to the ARRIVE guidelines and were strictly in accordance with the National Research Council Guidelines for the Care and Use of Laboratory Animals.

## Conflicts of Interest

The authors declare no conflicts of interest.

## Supporting information



Supporting Information

## Data Availability

The data that support the findings of this study are available from the corresponding author upon reasonable request.
