# Supplementary material for: Rational Design, Synthesis, and Biological Assessment of Potential Indole‐Capped HDAC6 Inhibitors for Gastric Cancer Suppression
Source: MedComm (2020). 2025 Mar 20;6(4):e70158. doi: 10.1002/mco2.70158 (PMC11923385; doi:10.1002/mco2.70158)
Supplement: Supplementary file 1 — Supporting Information [file MCO2-6-e70158-s001.docx]

**Supplementary information**

Rational Design, Synthesis and Biological Assessment of Potential Indole-capped HDAC6 Inhibitors for Gastric Cancer Suppression

Ya Gao^1^, Hai-Qian Nie^1^, Hong-Min Liu^1*^, Xin-Hui Zhang^2*^, Li-Ying Ma^1*^

^1^State Key Laboratory of Esophageal Cancer Prevention & Treatment; Key Laboratory of Advanced Drug Preparation Technologies, Ministry of Education of China; Key Laboratory of Henan Province for Drug Quality and Evaluation, Henan Province; Institute of Drug Discovery and Development; School of Pharmaceutical Sciences, Zhengzhou University, 100 Kexue Avenue, Zhengzhou, Henan 450001, China

^2^School of Biological Engineering, Henan University of Technology, Zhengzhou, Henan 450001, China

* Correspondence: maliying@zzu.edu.cn (L.-Y. Ma), zhangxinhui@haut.edu.cn (X.-H. Zhang), liuhm@zzu.edu.cn (H.-M. Liu)

**Methods and Materials**

# *1. General*

During the chemical synthesis, all of the solvents and reagents were bought from commercial companies. The chemistry reactions were monitored by TLC and LC-MS (Water, Milford, MA). Column chromatography was performed at medium pressure with silica gel (200-300 mesh). Spectra data of ^1^H NMR and ^13^C NMR were obtained on Bruker AVANCE III 400 M spectrometer (Bruker Instruments, Inc.). The mass spectrum data was recorded by Waters ACQUITY UPLC H-Class ACQUITY QDa (Water, Milford, MA), with electrospray ionization (ESI), C18 column; column size 2.1 mm × 50 mm; mobile phase 10%-95%, acetonitrile-water-0.1% formic acid served as a binary gradient mobile phase with a flow rate of 0.5 mL/min. The melting points of target compounds were tested by the WRS-1A digital melting point apparatus. The ^1^H, ^13^C NMR and HRMS spectra were shown in Supplementary Material.

# *2. Enzymatic assay*

The HDAC6 substrate Ac-Lys-AMC (Chinapeptides, China), human recombinant protein HDAC6 (100 ng/mL) (BPS bioscience, USA), and the test compounds were incubated together at 37°C in an assay buffer. The assay buffer consisted of 25 mM HEPES (pH 7.5), 100 mM NaCl, 2.5 mM KCl, 0.005% Tween-20, 1 mM MgCl_2_, and 0.1 mg/mL bovine serum albumin. After incubation for 1 hour, trypsin was added to release the fluorescent fragment.

# *3. BLI assay*

BLI experiments were conducted using an Octet Red 96 instrument (ForteBio). Streptavidin-coated biosensors (SA, ForteBio) were utilized, and biotinylated HDAC6 protein (50 μg/mL) was immobilized on the biosensor surface. The immobilization levels typically exceeded 0.3 nm. Ligand-loaded SA biosensors were then exposed to various concentrations of compound **10n**. The dissociation constants for the interactions between HDAC6 and compound **10n** were determined by plotting the increase in BLI response as a function of protein concentration.

# *4. Western blot*

Following the indicated treatment, cells were collected and lysed using the recommended amount of radio immunoprecipitation assay (RIPA) buffer from KMT Bio, China, according to the provided instructions. The protein concentration in the lysate was determined using a bicinchoninic acid (BCA)-based protein quantification kit from KMT Bio, China. Subsequently, the samples were denatured in the presence of a 6× loading buffer from KMT Bio, China. Equal amounts of denatured protein samples were subjected to sodium dodecyl sulfate-polyacrylamide gel electrophoresis (SDS-PAGE), and the target protein was then transferred to a nitrocellulose membrane using the wet transfer method with Pall, USA membranes. The membrane was incubated with 5% non-fat milk at 37°C for 1 hour to block non-specific binding, followed by an overnight incubation at 4°C with the recommended concentration of the primary antibody. Afterward, the membranes were washed three times with PBST (PBS buffer with 0.1% Tween-20) for 10 minutes each. The secondary antibody was then appropriately diluted and incubated with the membrane at room temperature for 1 hour. Finally, the membranes were washed three more times with PBST for 10 minutes each, and the protein bands were visualized using an ECL chemiluminescence detection kit from ThermoFisher, USA.

# *5. In vivo studies*

Six male ICR mice were divided into two groups, receiving compound **10n** via intravenous and oral administration, respectively. The intravenous injection group had blood samples collected at 5 min, 15 min, 30 min, 1 h, 2 h, 4 h, 6 h, 8 h, and 24 h post-administration; the oral group had blood samples collected at the same time points. Approximately 0.05 mL of blood was collected for each sample. The concentration of compound **10n** in mouse plasma samples was determined using LC-MS/MS method and pharmacokinetic parameters were calculated using WinNolin software.

Six-week-old 615 mice (male) were procured from SJA laboratory animals in Hunan, China, and acclimated to a pathogen-free environment for one week before the commencement of the experiment. Subsequently, 5 × 10^5^ of gastric cancer cell MFC were subcutaneously administered into the right flank of the mice. Tumor volume was determined using the formula length × width^2^/2. Upon reaching a tumor volume of 50-100 mm³, 24 mice were stratified into four groups and administered oral doses of 0, 10, or 20 mg/kg of compound **10n**, as well as 20 mg/kg of SAHA, delivered once daily, separately. Following 14 days, the mice were euthanized, and their tumors and vital organs were dissected for analysis. Paraffin sections measuring 4 µm in thickness were subjected to deparaffinization and subsequently treated with 3% hydrogen peroxide to inhibit endogenous peroxidase activity. Following this, slides were prepared to prevent nonspecific antibody binding and were then exposed to primary antibodies targeting HDAC6, Ac-tubulin, Ki-67, and Tunnel. The mice involved in the study were maintained in specific pathogen-free environments, and the experimental procedures were approved by the Ethics Committee of the Zhengzhou University Health Science Centre. The animal experimentation ethics number is ZZU-URIB-2024-O-109. All experimental animals adhered to the ARRIVE guidelines and were strictly in accordance with the National Research Council Guidelines for the Care and Use of Laboratory Animals.

# *6. Molecular docking*

The 3D structures of the compounds for docking were built using MOE 2019, and energy minimization was processed using the force field AMBER 10: EHT. The X-ray crystal structures of HDAC6 (PDB code 5EF7, 1.9 Å) were obtained from the PDB database, and prepared with the QuickPrep module of MOE 2019 using the default parameters. The co-crystalized ligand of the protein was used to define the active site for docking. The default Triangle Matcher placement method was employed for docking, and GBVI/WSA dG scoring function was used to assess the free energy of binding of compound **10n** from a given pose and rank the final poses.

# *7. Statistical analysis*

The data were plotted and analyzed using GraphPad Prism 8.0 (USA). Statistical significance was evaluated using the t-test or one-way analysis of variance (ANOVA). A significance level of P < 0.05 was considered indicative of a significant difference.
